# Cognitive recovery after critical illness: comparing English and Spanish-speaking survivors

**DOI:** 10.1186/s13054-026-06126-2

**Published:** 2026-06-22

**Authors:** Ana Lucia Fuentes, Charlotte C. Ellberg, Faizah Shareef, Mark Rolfsen, Amy Bellinghausen, Jamie N. LaBuzetta, Atul Malhotra, Robert L. Owens

**Affiliations:** 1 Division of Pulmonary, Critical Care and Sleep Medicine, University of California, San Diego, CA USA; 2 Department of Medicine, University of California, San Diego, CA USA; 3 Division of Allergy, Pulmonary and Critical Care Medicine, Vanderbilt University, Nashville, TN USA; 4 Brain Dysfunction and Survivorship (CIBS) Center, Critical Illness, Nashville, TN USA; 5 Department of Neurosciences, Division of Neurocritical Care, University of California, San Diego, CA USA

Delirium is a syndrome of acute brain dysfunction that is common in the ICU and is a strong predictor of post-ICU cognitive impairment [1]. Despite growing recognition of these sequalae, patients with limited English proficiency(LEP) have been largely excluded from large-scale studies evaluating post-ICU cognitive recovery. This knowledge gap is concerning given emerging evidence of language-based differences in delirium care, which may place patients with LEP at risk of poor cognitive outcomes [2, 3]. To begin to address this gap, we compared the cognitive performance between English and Spanish-speaking ICU survivors 9-months after hospital discharge.

Enrollment occurred between August 2024-January 2025. We identified eligible patients through daily review of the electronic health record(EHR). Eligible participants were English or Spanish-speaking adults admitted to two mixed ICUs. We excluded patients with moderate-to-severe cognitive impairment, those unable to complete delirium assessments, and those admitted for active seizures or alcohol withdrawal. After obtaining informed consent, we collected demographic data from participants and clinical characteristics from the EHR. We also assessed delirium using the Confusion Assessment Method for the ICU(CAM-ICU) and sedation using the Richmond Agitation-Sedation Scale(RASS) daily for three consecutive days after enrollment. Assessments were completed by trained bilingual members of the research team within 2 h of bedside provider evaluations (usually 7–9 AM). Additional protocol details have been previously described [3]. We then contacted participants 9 months following hospital discharge and completed cognitive assessments using the Telephone Interview for Cognitive Status(TICS). Scores < 26 indicated cognitive impairment based on established guidelines. All assessments were conducted in the patient’s preferred language.

Of 142 patients enrolled (71 English-speakers,71 Spanish-speakers), 43 died during hospitalization and 21 died before the 9-month follow-up. Of 78 survivors, 67 completed cognitive evaluation (34 English-speakers,33 Spanish-speakers; 1A). English-speakers had higher median(IQR) TICS scores than Spanish-speakers (29[6] vs. 25[6], *p* < 0.05), and lower rates of cognitive impairment (34% vs. 55%,*p* < 0.05; Table 1B). In unadjusted linear regression, English-speakers scored 4.1 points higher on TICS than Spanish-speakers. This difference remained after adjustment for age, sex, education, cognition, and illness severity (3.6 points), and was modestly attenuated after further adjustment for delirium or sedation (3.0 and 3.4 points, respectively; Table 1C).

**Table 1:** Patient demographics and cognitive outcomes in ICU survivors completing follow up

**Panel A. Patient demographics and characteristics**								
**Patient characteristics**				**English-speakers** **(n=34)**			**Spanish-speakers** **(n=33)**	
Age mean (SD)				59 (15)			60 (15)	
Biological Sex n (%)								
Female				17 (50)			14 (42)	
Race n (%)								
White				21 (60)			8 (28)	
Black or African-American				4 (13)			-	
Asian				1 (3)			-	
Native Hawaiian or Other acific islander				-			-	
American Indian or Alaskan Native				-			-	
Mixed				8 (24)			24 (72)	
Ethnicity n (%)								
Hispanic or Latino				10 (30)			33 (100)	
Education n (%)								
Less than high school				2 (6)			11 (33)	
High school graduate				8 (24)			15 (46)	
Some college				8 (24)			1 (3)	
Bachelors degree or higher				16 (46)			6 (18)	
Prior diagnosis of mild cognitive impairment* n (%)				2 (1)			1 (1)	
Mechanical Ventilation n (%)				16 (47)			18 (54)	
Delirium, CAM-ICU Positive n (%)				11 (32)			15 (45)	
APACHE II Score mean (SD)				18 (7)			18 (8)	
ICU LOS median (IQR)				7 (12)			9 (14)	
Hospital LOS median (IQR)				14 (16)			18 (20)	

**Panel B. Cognitive outcomes by language**								
**Outcome**	**English-speakers**		**Spanish-speakers**		**Chi-square statistic (χ²)**			**P value**
Cognitive Impairment (TICS < 26), n (%)	10 (34)		18 (55)		4.1			<0.05

**Panel C. linear regression models of TICS score by language **								
**Model**		**Β**†** (95% confidence interval)**				**P value**		
Unadjusted		4.1 (0.7-6.1)				<0.05		
Adjusted Model (age, sex education, APACHE II)		3.6 (1.1-6.0)				<0.05		
Adjusted Model + delirium (CAM-ICU positive)		3.0 (0.8-5.5)				<0.05		
Adjusted Model + sedation (RASS≤ -3)		3.4 (1.4-5.5)				<0.05		

* Prior cognitive impairment was determined by review of the electronic health record

† β coefficients represent the mean difference in Telephone Interview for Cognitive Status (TICS) score for English compared with Spanish-speaking participants

Our findings suggest that Spanish-speaking ICU survivors may experience worse long-term cognitive outcomes than English-speakers. This finding is likely multifactorial, but disparities in ICU care delivery may play an important role. Our work and that of others has shown that patients with LEP, including Spanish-speakers, experience deeper sedation, higher rates of missed delirium, and lower implementation of delirium prevention strategies (e.g., occupational/physical therapy)—all of which may contribute to greater delirium burden versus English-speakers [2, 3]. Given the well-established association between delirium and cognitive impairment, disparities in these care practices may represent one mechanism underlying the cognitive differences we observed. In support of this potential pathway, adjustment for delirium and depth of sedation(RASS) attenuated the association between language and cognitive outcomes. However, differences persisted after adjustment for these factors, suggesting that additional unmeasured variables (e.g.,cumulative sedation/analgesia) may also contribute.

Differences in cognitive outcomes may also reflect greater baseline vulnerability and differences in ICU recovery among Spanish-speaking survivors, and more broadly, patients with LEP. Patients with LEP often experience reduced healthcare access, more miscommunication with providers, and lower treatment adherence than English-speakers [2–5], factors that may influence both pre-ICU vulnerability and recovery after critical illness. Although we focused on language, broader social determinants of health(e.g.,socioeconomic and insurance status) are also likely relevant and may contribute to adverse cognitive outcomes through differences in healthcare access before and after ICU admission, including access to ICU recovery clinics. While efforts like telemedicine have aimed to improve accessibility, financial, technological, and logistical barriers may still limit access for some patients.

Interestingly, similar disparities in cognition have been observed outside of the ICU, where Hispanic populations have a higher risk of Alzheimer’s disease and related dementias(ADRD) versus non-Hispanic White populations [4]. These disparities likely reflect social, environmental, and genetic factors, some of which may overlap with post-ICU cognitive impairment. While we excluded patients with moderate-severe cognitive impairment, future research should determine whether language-based disparities throughout critical illness and recovery similarly affect this particularly vulnerable population. These patients may face additional challenges navigating ICU recovery care (e.g., competing medical appointments, limited transportation, reduced ability to participate in recovery interventions), potentially compounding an already elevated risk of worsening cognitive impairment.

Our findings highlight the need for rigorous research in diverse ICU populations to better understand factors contributing to disparities in cognitive outcomes before, during, and after critical illness. Although some factors are non-modifiable, disparities in ICU care delivery represent important modifiable targets. For example, language-concordant care and family engagement have been shown to reduce disparities in ICU care delivery by improving delirium detection and reducing restraint use among Spanish-speakers [2, 3]. Additionally, more objective approaches to delirium and sedation assessment(e.g.,biomarkers, EEG-based assessments) are being developed that may bypass communication barriers and improve delirium detection and reduce oversedation. Improving ICU care delivery may ultimately help reduce disparities in long-term cognitive outcomes.

These findings should be interpreted with caution. Although TICS has been translated into Spanish, formal cross-cultural validation remains limited, and cognitive screening tools may misclassify impairment when applied across linguistic and cultural groups without rigorous cross-cultural adaptation and validation [5]. Some observed differences may therefore reflect measurement limitations rather than true disparities in cognitive function. Our modest sample size further limits statistical power and the ability to fully adjust for confounding. These findings should be considered preliminary and hypothesis-generating.

In conclusion, English and Spanish-speaking ICU survivors demonstrated meaningful differences in long-term cognitive outcomes, raising concern that inequities across the course of critical illness may have lasting consequences. Larger, more inclusive studies are needed to confirm these findings, better characterize the mechanisms driving disparities, and support the delivery of more equitable critical care.

## Data Availability

No datasets were generated or analysed during the current study.
